# Contrast-enhanced endoscopic ultrasound-guided additional ethanol ablation after early detection of occult residual pancreatic insulinoma

**DOI:** 10.1055/a-2817-2984

**Published:** 2026-03-09

**Authors:** Ryo Sugiura, Masaki Kuwatani, Kazumichi Kawakubo, Shoya Shiratori, Soichiro Oda, Katsuma Nakajima, Naoya Sakamoto

**Affiliations:** 1 163693 Department of Gastroenterology and Hepatology, Hokkaido University Hospital, Sapporo, Japan

The usefulness of endoscopic ultrasound (EUS)-guided ethanol ablation (EUS-EA) for small non-functional pancreatic neuroendocrine neoplasms and insulinomas has been reported 1 2 . Even when sufficient ablation appears achieved, additional ablation is sometimes required within 3–5 days 1 . However, additional treatment can be difficult when residual lesions are not identifiable on conventional EUS. We report a case with occult residual insulinoma after EUS-EA that was undetectable on conventional EUS but identified by contrast-enhanced EUS (CE-EUS), enabling additional ethanol ablation.

**Fig. 1:**
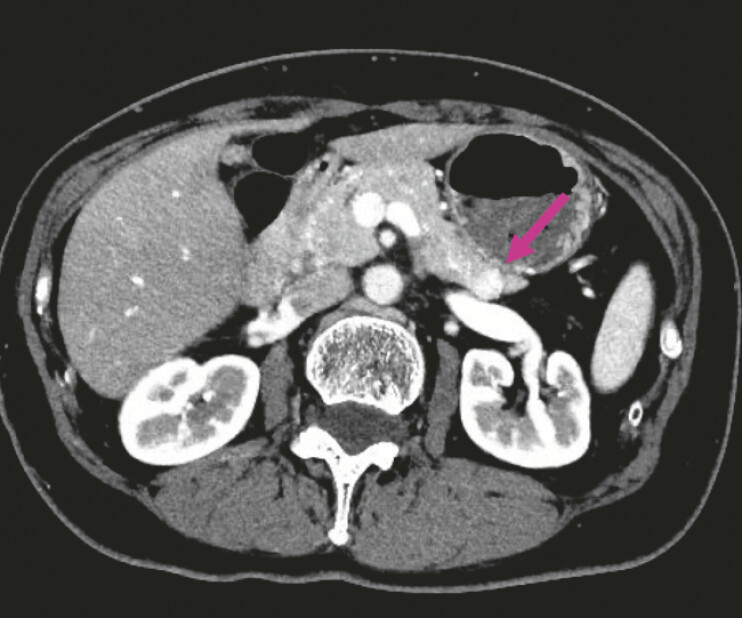
Contrast-enhanced computed tomography demonstrated a 10-mm tumor (arrow) in the pancreatic tail with early enhancement.

**Fig. 2:**
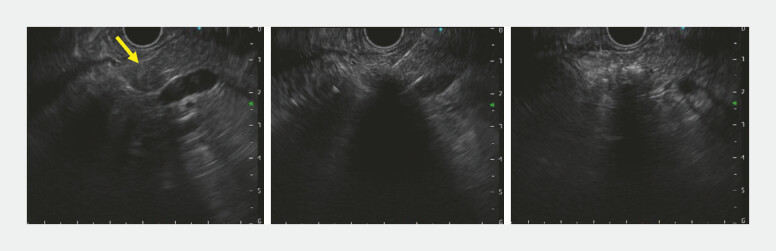
(Left) EUS showed a nearly round pancreatic tumor (arrow). (Middle) EUS-guided ethanol ablation was performed. (Right) The tumor showed uniform hyperechoic changes after EUS-guided ethanol ablation. EUS, endoscopic ultrasound.

**Fig. 3:**
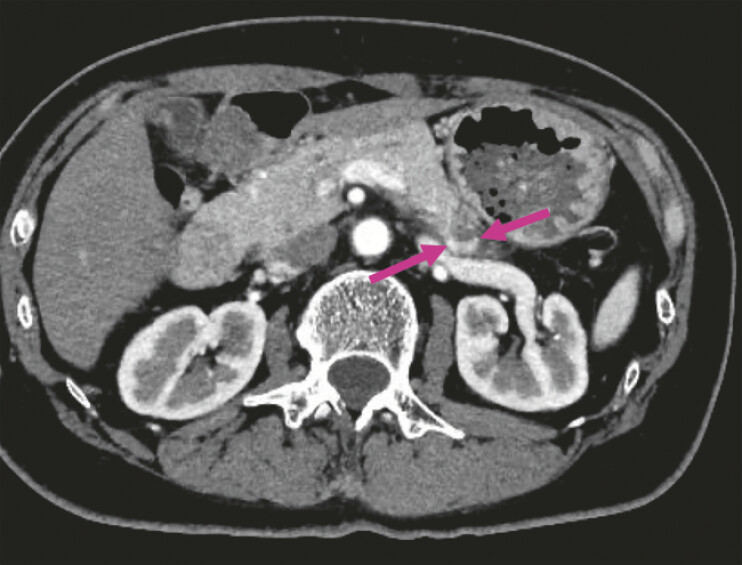
An early-enhancing area (arrow) at the lesion margin on contrast-enhanced computed tomography performed 3 days after the procedure.

**Fig. 4:**
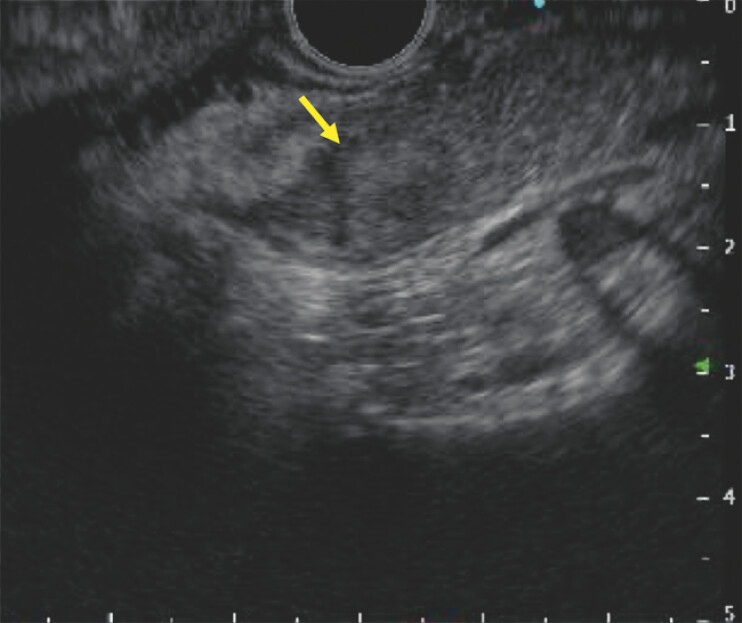
EUS demonstrated a heterogeneous echo pattern in the tumor area (arrow) after the initial procedure, making localization of residual tumor difficult. EUS, endoscopic ultrasound.

**Fig. 5:**
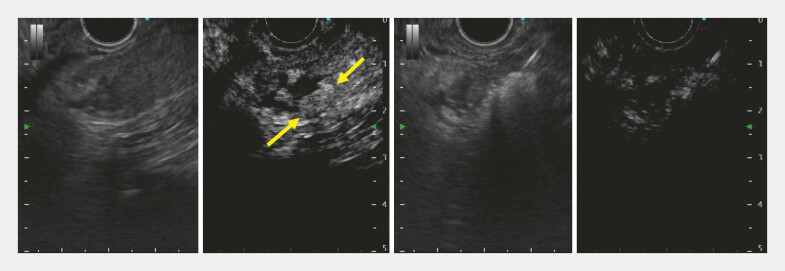
(Left) Contrast-enhanced endoscopic ultrasound visualized the residual tumor (arrow). (Right) Additional endoscopic ultrasound-guided ethanol ablation was performed on the residual tumor.

A 57-year-old woman was referred for an insulinoma causing recurrent hypoglycemia. Contrast-enhanced computed tomography (CE-CT) showed a 10-mm tumor in the pancreatic tail with early enhancement ( Fig. 1 ). EUS-EA was performed. The tumor was punctured with a 25-gauge needle filled with undiluted ethanol (99.5% v/v) without a stylet, and 1.7 mL was injected until the lesion became hyperechoic ( Fig. 2 ). The needle was left in place for 1 minute and withdrawn. CE-CT 3 days later demonstrated an early-enhancing area at the lesion margin, suggestive of residual tumor ( Fig. 3 ). As the patient continued occasional hypoglycemia, additional EUS-EA was planned. Conventional EUS showed a heterogeneous echo pattern, making localization difficult ( Fig. 4 ). After intravenous perflubutane injection, the ablated area showed no enhancement, whereas the residual tumor became clearly visible on CE-EUS, allowing additional ethanol ablation with 1.0 mL ( Video 1 ; Fig. 5 ). CE-CT 3 months later revealed no residual tumor, and no further hypoglycemic episodes were observed.

Contrast-enhanced endoscopic ultrasound-guided additional ethanol ablation performed after detection of an occult residual pancreatic tumor.Video 1

Several reports have demonstrated the utility of CE-EUS for early evaluation of residual tumor after EUS-guided radiofrequency ablation of pancreatic neoplasms 3 4 , whereas, to our knowledge, no such reports exist for EUS-EA. Therefore, CE-EUS should be considered when residual tumor after EUS-EA is difficult to delineate. Treatment options for insulinomas include surgery, medical therapy, or minimally invasive approaches such as EUS-EA, which can be considered a less invasive alternative for selected small insulinomas.

Endoscopy_UCTN_Code_TTT_1AS_2AD Endoscopy_UCTN_Code_TTT_1AS_2AI
